# Empyema of the Cavity of the Right Upper Jawbone in the Horse

**Published:** 1898-03

**Authors:** 


Empyema of the Cavity of the Right Upper Jawbone in the Horse. By Schuemacher.—A valuable work-horse, four and a half years old, had been for a month not so lively as usual—often hung its head to the ground, and ate less than in common; yet it was able to work until November 22, 1896, when it came under my care. It showed great faintness, difficulty in breathing, with contraction of the flanks, and groaned for sometime after lying down. There was a slimy, mattery running at the nose, principally on the right side; with it all there was still some desire to eat; the temperature, 39.6°–40.5° C.; pulse 62; respiration 28-35 to the minute. On the 24th symptoms of acute pleuro-pneumonia on both sides were present, which, in spite of energetic treatment, caused death on the 28th of November.

The post-mortem examination, made on the same day under the auspices of the Baden Insurance Company, gave the following result: The stomach and intestinal tract, in appearance, mucous membrane, and contents, were normal; likewise the peritoneum, liver, and spleen. Both kidneys, especially the right, were considerably enlarged; the layer-substance muddy and swollen, the marrow-substance reddish-brown; in the kidney-chambers about 3 gr. of purulent slime. In the abdominal cavity about 2½ litres of unclear, reddish-yellow fluid filled with fibrin coagulations; the pleura costalis, especially on the right, coated with a yellow, flaky layer of fibrin, in places deeply reddened. In the windpipe a foamy, filthy liquid; the mucous membrane of the same and of the larynx were swollen, very red, and juicy. In both lobes of the lungs, and especially at the points, thickened tissue; on cutting through, purulent gangrenous congestions and caverns filled with decayed tissue were found.

To find out the cause of this purulent, gangrenous inflammation of the lungs, the skull was sawed through, for it was not an ordinary case of pneumonia. On opening the cavity of the right upper jaw it was found to be filled with a yellow, crumby pus; the thin lamella, which normally divides the cavity into two chambers, was wanting; the periosteum of the surrounding bones was 1.5–2 mm. thick, easily removed, bluish-red, and very sappy; the roots of the molar teeth 2 and 3 were carious; the bony edges of the apertures leading to the right nasal canal looked as if they were eaten through by caries.

The course of the disease is probably to be explained as follows: Originally a purulent alveolar periostitis and caries of the root of the second and third molars; irruption of the pus into the cavity of the right upper jawbone, and a continuous purulent inflammation of the mucous membrane of this cavity and the periosteum, with caries of the surrounding bones; then the formation of an abscess; further, a flow of pus through the aperture sinus maxillaris into the nasal cavity; inspiration of the pus through the windpipe into the lungs, causing here a purulent, gangrenous pneumonia, and finally pleuritis; secondary metastatic nephritis, as a general infectious phenomenon.

The condition in the cavity of the upper jaw and its surroundings, revealed by the post-mortem examination, shows that at times a pneumonia, whose cause cannot be explained, or is wrongly attributed to a cold, is to be attributed to a pathological process in the upper channels of respiration and its environment.

In conclusion, it is worthy of mention that during life no swelling or other change was noticeable in the region of the upper jaw which could have suggested an operation (trephining) as a possible remedy.—*Idem.*

---

THE increased attention to riding is now noted in the scarcity of good saddle-gaited horses. Members of riding clubs over the country, especially in the large cities, are much more interested in the mannering and gaiting of horses, and to better appreciate the best form are securing the services of veterinarians and others through series of lectures in their clubs. New York has already been edified by a series of lectures by Prof. R. S. Huidekoper, and Boston's riding club follows in the same direction.

